# *Tecomella undulata* under threat: The impact of climate change on the distribution of a valuable tree species using a machine learning model

**DOI:** 10.1371/journal.pone.0326609

**Published:** 2025-07-09

**Authors:** Ehsan Ghafouri, Gholamabbas Ghanbarian, Artemi Cerdà, Saeideh Ghafouri

**Affiliations:** 1 Department of Natural Resources and Environmental Engineering, School of Agriculture, Shiraz University, Shiraz, Iran; 2 Department of Geography, Soil Erosion and Degradation Research Group, University of Valencia, Valencia, Spain; University of Uyo, NIGERIA

## Abstract

Climate change has emerged as a significant driver of biodiversity loss, with profound implications for species distribution. This study assessed the current and future distribution of *Tecomella undulata* (Desert teak), an economically and medicinally valuable species facing threats from climate change. MaxEnt model, built using 44 occurrence points and environmental data including bioclimatic factors and Digital Elevation Model (DEM), demonstrated an impressive Area Under the Curve (AUC) value of around 0.91 and a True Skill Statistic (TSS) value of 0.79, indicating excellent predictive performance. Temperature seasonality (Bio4) emerged as the most crucial variable, contributing 35.9% to the modeling, followed by the mean temperature of the wettest quarter (Bio8) and precipitation seasonality (Bio15). The habitat suitability maps showed a strong presence of *T. undulata* in the southern regions of Iran, with Fars and Bushehr provinces being particularly conducive to its growth. Future projections under Shared Socioeconomic Pathways (SSP) scenarios SSP245 and SSP585 for 2030, 2050, 2070, and 2090 suggested a decline in suitable habitats for *T. undulata*, with high-suitability areas projected to decrease by up to 98% and unsuitable habitats predicted to increase. The study underscores the urgency for tailored conservation measures to mitigate the impacts of climate change on this valuable species.

## Introduction

The loss of biodiversity, driven by habitat destruction, resource overexploitation, pollution, and invasive species [1,2], underscores the need for green technology innovation in sectors like agriculture and industry, which are major contributors to environmental emissions [3,4]. While climate change is not currently the primary driver of biodiversity loss, its impact is increasing and is expected to become a major contributor in the near future [5,6]. The implications of this decline are far-reaching, impacting not only the intricate web of life on Earth but also posing direct and indirect consequences for humanity [7].

Climate change, characterized by shifts in temperature and precipitation patterns, is already instigating range shifts in various species, a trend projected to intensify in the future [8]. These climatic alterations disrupt the delicate balance that shapes species distribution, affecting their reproductive success and overall viability. The fragmentation of habitats, driven by global warming, is pushing some species towards extinction while forcing others to migrate in search of more suitable climates [9,10]. The urgency of this situation is underscored by the Intergovernmental Panel on Climate Change’s (IPCC) sixth assessment report, which reveals alarming trends in global temperature rise and paints a concerning picture of future climatic conditions [11].

Understanding the intricate relationship between climate change and species distribution is paramount. As species respond to climatic shifts through changes in their geographic range, phenology, and other ecological attributes, we witness fluctuations in their abundance and, in some cases, their complete disappearance [12]. Therefore, the ability to predict the impact of future climate change on species distribution is essential for the development of effective conservation strategies.

Technological advancements in machine learning, ecological theory, and geographic information systems (GIS) are revolutionizing the fields of ecology and conservation, offering novel tools to address these challenges [13,14].

Species distribution modeling (SDM) stands at the forefront of these innovations, enabling researchers to generate predictive maps of species distribution based on their known locations and environmental data. By unraveling the complex interplay between abiotic environmental factors and species occurrence, SDMs provide invaluable insights into the ecological niches of species [14–16]. As modeling algorithms undergo continuous refinement, the use of habitat utility models is becoming increasingly prevalent in the fields of environmental science, biogeography, conservation biology, and species management [17].

Among the various SDM algorithms, MaxEnt has garnered widespread recognition for its ability to produce accurate predictive models [14,17–19]. This modeling approach has proven effective for various species, but it holds particular value for endangered species. It consistently outperforms other presence-only models in generating accurate predictive models, especially when dealing with limited input data [19].

The urgency for safeguarding endangered species becomes particularly pronounced in harsh climates and areas characterized by low biodiversity. The Desert teak (*Tecomella undulata*), a deciduous shrub or small tree belonging to the Bignoniaceae family, exemplifies such a species. This plant, also referred to as Marwar teak or Devil’s pomegranate (Anar Sheitan in Persian), holds significant economic and medicinal value [20]. There are usually small, pure masses growing along rivers and in relatively flat areas, its global distribution spans South Asia, including India, Pakistan, southern Iran, and the Arab states of the Persian Gulf, extending into North Africa [21]. Within southern Iran, it finds its niche in the provinces of Fars, Kerman, Hormozgan, Bushehr, and Sistan-Baluchestan. *T. undulata* is not only admired for its vibrant yellow, red, and orange flowers but also highly valued in the pharmaceutical industry for its rich content of mucilage, vitamins, phenolic compounds, and fatty acids [22].

This species exhibits a remarkable adaptability to thrive on stabilized sand dunes, tolerating extreme temperature fluctuations and arid conditions with minimal rainfall [20]. The plant's extensive root system forms a strong network on the surface of the soil, making it ideal for creating windbreaks, protecting the environment, and preventing quicksand movement and desertification. It also provides shade for desert animals and nesting sites for birds [23]. A decline in *T. undulata* populations could have cascading effects on local biodiversity, potentially decreasing species richness and ecosystem resilience. The loss of this species could also exacerbate land degradation. This tree species is widely used in agroforestry because it can survive in extreme conditions. However, it grows slowly and there is no easy way to propagate it quickly [20].

To ensure the effective management and conservation of *T. undulata*, a comprehensive understanding of its habitat requirements under present and future climate scenarios is essential [18,24]. Despite *T. undulata*’s importance, there is a critical knowledge gap regarding how climate change will impact its future distribution. While some studies have modeled its distribution [14,15,18], few have projected how shifting climate patterns will affect its suitable habitats in the future, especially in the context of shared socioeconomic pathways (SSPs), which explore the combined effects of climate and socioeconomic factors. Also, the effect of climate change on the distribution of this species in Iran has not been investigated.

This study aims to address this critical knowledge gap by employing the MaxEnt model to:

1. Predict the current and future distribution of *T. undulata* in Iran under different climate change scenarios.
2. Identify the key environmental factors influencing its distribution.
3. Provide a scientific foundation for the conservation and management of this valuable species in the face of climate change.

By elucidating the potential impacts of climate change on *T. undulata*’s distribution and pinpointing the environmental variables that shape its habitat suitability, this research will contribute valuable insights to guide conservation efforts and ensure the long-term survival of this ecologically and economically significant species.

## Materials and methods

### Study area

**Fig 1:**
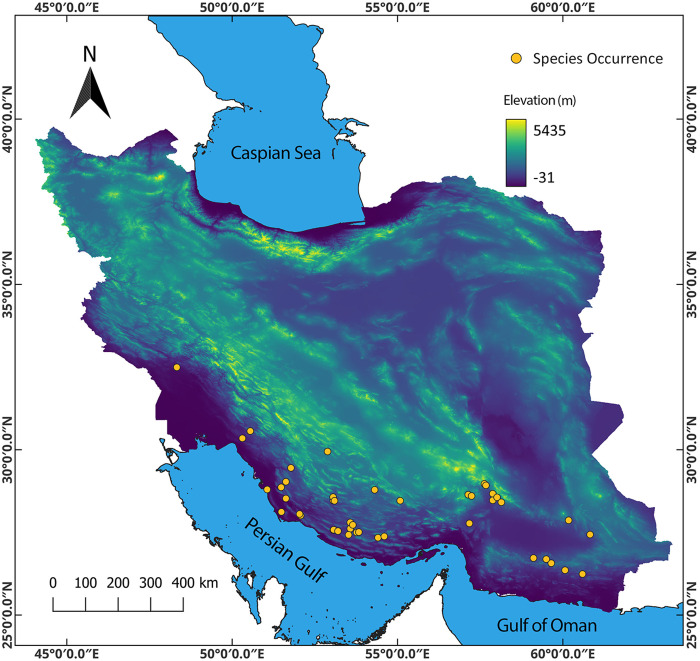
The study area and occurrence points of Desert teak. Map was generated using QGIS 3.4.1 (https://www.qgis.org).

The study area includes the entire country of Iran, and it is located between 25 and 40 degrees north latitude and 44 and 64 degrees east longitude (Fig 1). This country is located in three phytogeographic regions: the Irano-Turanian, the Saharo-Sindian, and the Euro-Siberian. It also covers two global biodiversity hotspots: Irano-Anatolia and the Caucasus [25]. Iran boasts considerable species richness and biodiversity across various taxonomic groups. Despite its under-representation in biodiversity-climate warming studies, Iran holds significance as a biodiversity hotspot. The country's rich habitat diversity allows for a broad spectrum of species to thrive within its borders [26]. Iran is a vital region in the Middle East for preserving biodiversity, and its ecosystems contain around 8,000 plant species [27].

Iran’s climate is predominantly arid and semi-arid, with most of its relatively limited annual precipitation occurring between October to April. Iran features a diverse range of weather patterns across its regions, marked by notable yearly precipitation differences from 120 to 2000 mm and temperature fluctuations between −20 and 50 °C [28]. Apart from the higher mountain valleys of the Zagros and Caspian coastal plain, most regions receive an average annual precipitation of 250 mm or less. Iran’s average elevation is 1257 meters above sea level, with variations ranging from approximately −31 meters to over 5425 meters. Two primary mountain ranges, the Alborz, stretching from northwest to northeast, and the Zagros, extending from northwest to southeast, enclose the vast Iranian plateau, an arid expanse encompassing rugged deserts and salt flats known as Kavir [19].

### Species occurrence record

We obtained 52 occurrence points (presence-only) for *T. Undulata* from extensive field surveys conducted in 2021–2023, literature review, and the assistance of environmental staff, environmental activists, and local people. Permits were not required to collect species presence points at certain sites. The geographical coordinates of the records were checked and refined using Google Earth, and then saved in CSV format.

The accuracy of the prediction is enhanced by the spatial autocorrelation of sampling effort between the training and test data [29]. Spatial filtering is employed to mitigate the impacts of sampling biases and model over-fitting [30,31]. Therefore, spatial thinning of species occurrence was conducted using the spThin package [32] in R software. In this process, multiple presence locations in a given grid with a spatial resolution of about 1 km² were eliminated. Only one record was retained per grid. Finally, in total 44 occurrence points (Fig 1) were employed to build the model. The maps were prepared in the open source QGIS software.

### Environmental data

In this study, 19 bioclimatic factors (Table 1) at 30-arcsec resolution (~1 km2), derived from the WorldClim website, were used which include current climate conditions (www.worldclim.org). The current climate is the average data from 1970 to 2000 from the WorldClim version 2.1 [33]. Also, the Digital Elevation Model (DEM) derived from the SRTM elevation data was downloaded from the WorldClim website. We also downloaded bioclimatic variables from WorldClim for future climate scenarios (SSP24.5 and SSP58.5) in the 2030s (the average of period 2021-2040), 2050s (the average of period 2041-2060), 2070s (the average of period 2061-2080) and 2090s (the average of period 2081-2100), using the same resolution and the same bioclimatic layers used in the current conditions.

**Table 1:** Bioclimatic variables used in the study.

Variable	Code	Unit
Annual mean temperature	Bio1	°C
Mean diurnal range	Bio2	°C
Isothermality (Bio2/Bio7) (×100)	Bio3	Index
Temperature seasonality	Bio4	Index
Max temperature of the warmest month	Bio5	°C
Min temperature of the coldest month	Bio6	°C
Temperature annual range	Bio7	°C
Mean temperature of the wettest quarter	Bio8	°C
Mean temperature of the driest quarter	Bio9	°C
Mean temperature of the warmest quarter	Bio10	°C
Mean temperature of the coldest quarter	Bio11	°C
Annual precipitation	Bio12	mm
Precipitation of the wettest month	Bio13	mm
Precipitation of the driest month	Bio14	mm
Precipitation seasonality	Bio15	Index
Precipitation of the wettest quarter	Bio16	mm
Precipitation of the driest quarter	Bio17	mm
Precipitation of the warmest quarter	Bio18	mm
Precipitation of the coldest quarter	Bio19	mm

There are four main future climate paths known as shared socio-economic pathways (SSPs), which include SSP126, SSP245, SSP370, and SSP585. These SSPs help us understand how different social and economic developments might link to climate outcomes. According to these SSPs, we could see a global temperature rise of 3–5 °C by the year 2100. Specifically, the SSP126 envisions a sustainable, green future limiting warming to 3–3.5 °C; SSP245 takes a middle-of-the-road approach, with expected warming of 3.8–4.2 °C, as it continues current social, economic, and technological trends; SSP370 anticipates a future where countries prioritize regional development, leading to a temperature increase of 3.9–4.6 °C; finally, SSP585 predicts a scenario with unchecked economic and energy use growth, causing warming of 4.7–5.1 °C by the end of the century [34].

Between General Circulation Models (GCMs), we used ACCESS-ESM1–5, BCC-CSM2-MR, IPSL-CM6A-LR, and MRI-ESM2–0. These specific GCMs were chosen for their demonstrated ability to accurately forecast temperature and precipitation patterns within Iran, as evidenced by comparisons with observational data from various synoptic stations throughout the country. Previous research has utilized a range of methods for evaluating GCM performance. Recognizing the successful application of these GCMs in prior studies, along with their established use in the field, we selected these four models and applied them in an ensemble approach by averaging four GCMs [25,35–38]. These GCMs use the SSP126, SSP245, SSP370, and SSP585 scenarios of the shared socioeconomic pathways (SSPs) adopted by the IPCC 6th Assessment. SSPs build on existing national and regional development plans and trends to explore potential future socioeconomic landscapes. They are upgraded versions of representative concentration pathways (RCPs) that reflect the connection between socio-economic growth trends and climate change risks. [9,39]. We selected SSP245 and SSP585 to represent a range of plausible future climate pathways, from moderate to more extreme, allowing us to explore the potential impacts under different levels of greenhouse gas emissions and socioeconomic development. These scenarios are widely used in climate change research and provide a standardized framework for comparing results across studies [40–42].

To prevent model overfitting and multicollinearity between variables, we calculated the variance inflation factor (VIF) between the bioclimatic variables and DEM. We used the usdm package [43] and a threshold of 0.8 was set for VIF and removed highly correlated variables [44]. The remaining seven variables were Mean Diurnal Range (BIO2), Isothermality (BIO3), Temperature Seasonality (BIO4), Mean Temperature of Wettest Quarter (BIO8), Mean Temperature of Driest Quarter (BIO9), Precipitation of Driest Month (BIO14), Precipitation Seasonality (BIO15), Precipitation of Coldest Quarter (BIO19) and DEM.

### Model construction

We employed the Maximum Entropy (MaxEnt) algorithm [45] in R version 4.3.1, using the sdm package [46], to make predictions of *T. undulata* distributions. The model was run with 10,000 random background points. MaxEnt generates robust and precise predictive and distribution models comparable in accuracy to ensemble methods [47]. This well-established modeling approach offers a flexible response and strong transferability capabilities. Such attributes render it an appropriate model for projecting potential species distributions under varied environmental scenarios, making it a valuable asset for conservation and ecology-related applications [26].

We performed ten runs of our algorithm and employed a four-fold cross-validation technique to evaluate its accuracy. The dataset was randomly partitioned into four parts, with three parts used for model training and the remaining part held out for evaluation. We utilized the area under the receiver operating characteristic curve (AUC) [48] and true skills statistic (TSS) [49] methods to measure model performance. The ROC curve is not influenced by the prevalence of species, and the AUC indicates the model's ability to correctly distinguish between presence and absence points. The AUC value ranges from 0 to 1, with 0.5 indicating a random model. AUC values above 0.7 are considered valid and acceptable, while values above 0.9 indicate excellent results [26]. TSS scores range from +1 to −1, where a score closer to 1 signifies a more accurate model, while a score closer to zero or negative suggests a model performing no better than random chance [49]. We computed the relative variable importance metric by using Pearson correlation within the SDM package. Furthermore, we projected the future distribution of species using the models and considered both the SSP245 and SSP585 scenarios for 2030, 2050, 2070, and 2090. In addition, we classified the present and future suitability maps into four categories: unsuitable (< 0.1), low (0.1–0.4), medium (0.4–0.7), and high (> 0.7) suitability. To evaluate the impact of climate change on species distribution, we conducted comparisons between each future species distribution map with the current distribution maps.

## Results

### Importance of variables and model performance

**Fig 2:**
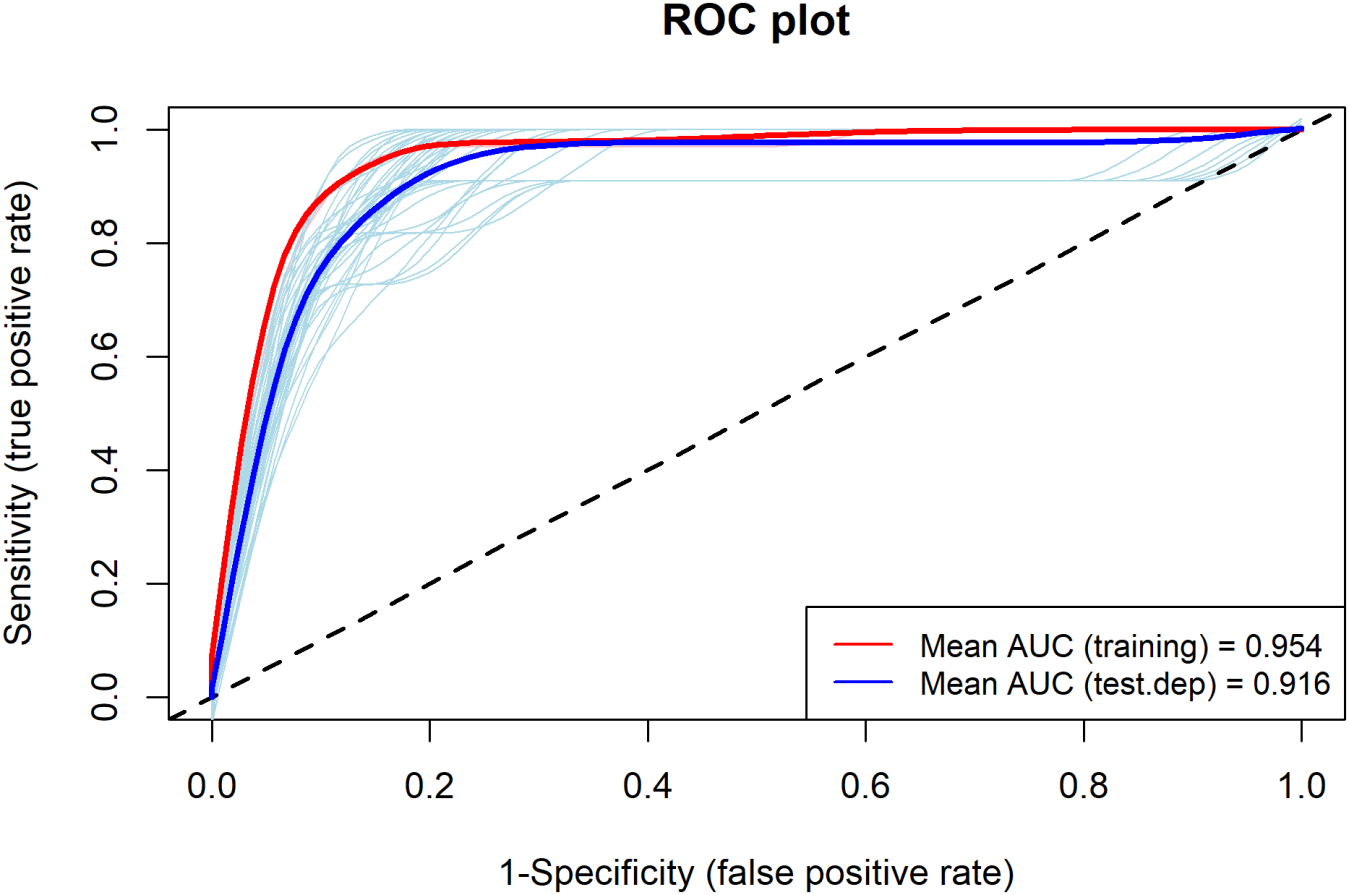
Area under the receiver operating characteristic curve (AUC) for MaxEnt model.

The MaxEnt model achieved excellent predictive performance (Fig 2) for the distribution of the *T. undulata* species, indicating that it can accurately predict the distribution of this species. The TSS value of 0.79 further confirms the model's good predictive accuracy.

**Fig 3:**
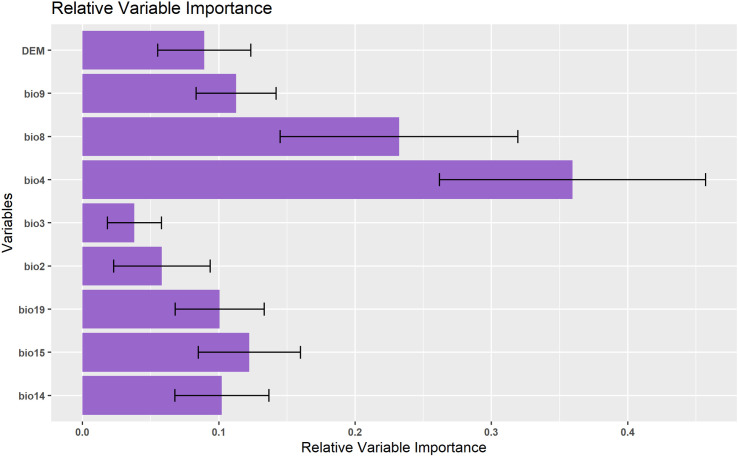
Relative Variable Importance.

The distribution model for *T. undulata* demonstrates that varied bioclimatic and geographical factors contribute substantially to its potential distribution (Fig 3). Temperature seasonality (bio4) holds prominent importance, contributing to 35.9% of the potential distribution of *T. undulata*. This points towards the species’ high sensitivity towards seasonal temperature fluctuations. Mean temperature of the wettest quarter (bio8) comes in second, accounting for 23.2% of the distribution. This shows the species’ affinity for warmer climates during the wettest quarters of the year. Precipitation seasonality (bio15) contributes 12.2%, signifying *T. undulata*’s dependence on precipitation fluctuations throughout the year. Mean temperature of the driest quarter (bio9) provides 11.3% of potential distribution insights, highlighting the species’ adaptability to temperature ranges in such periods. Precipitation of the driest (bio14) and coldest quarters (bio19) contribute 10.2% and 10.1% respectively, suggesting the species’ resilience during dry and cold periods. The digital elevation model (DEM) also serves as a significant factor with a contribution of 8.9%. This notably implies the species’ predisposition towards certain elevations. Mean diurnal range (bio2) plays a more moderate role, comprising 5.8% of the distribution. This demonstrates how daily temperature ranges can influence the species’ distribution. Finally, isothermality (bio3), contributing a marginal 3.8%, indicates the least effect on the species’ distribution.

From the contribution rates, we can observe that temperature factors including bio4, bio8, bio9, bio3, and bio2 collectively contribute a significant portion (80%) towards the potential distribution of *T*. *undulata*, underlining the species’ sensitivity towards temperature. On the other hand, the collective weight of precipitation factors such as bio15, bio14, and bio19 amount to 32.5%, proffering moisture’s role in the species’ geographic spread. Alongside bioclimatic influences, the species shows a marked preference for certain altitudes as per the Digital Elevation Model (DEM). These findings highlight the complex interplay of varied ecological factors in shaping the distribution of *T*. *undulata*.

### Predicting the geographical distribution of *T. undulata*

**Fig 4:**
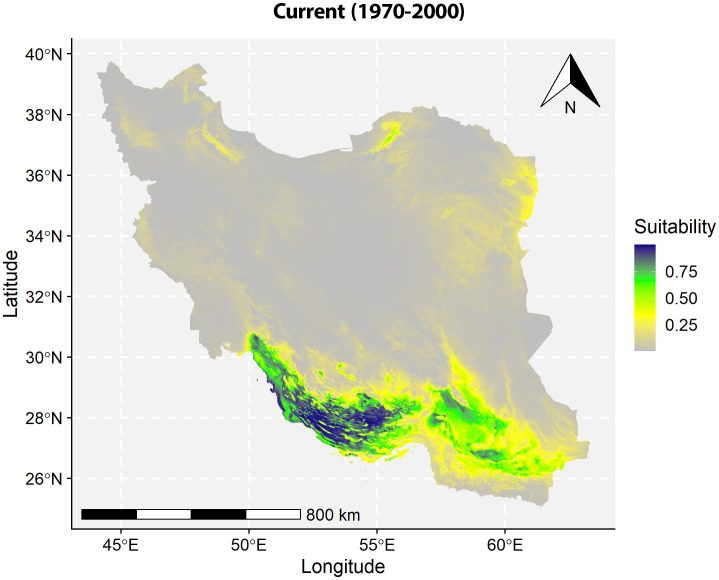
Habitat suitability of T. undulata. Map was generated using R 4.4.2 (https://www.r-project.org/).

The plant species *T. undulata* exhibits a marked affinity for the southern regions of Iran, with its natural geographical distribution primarily concentrated in this area. The provinces demonstrating the highest habitat suitability include Fars and Bushehr, which provide optimal environments for the species to thrive. Adjacent southern provinces, which comprise Kerman, Hormozgan, and Sistan-Baluchistan, also offer considerable levels of habitat suitability for *T*. *undulata* (Fig 4).

No suitable habitats were found for this species in most of the central and northern regions of the country. Only in some areas in the north and northeast of Iran, suitable habitats with medium to low suitability were observed. One such area can be seen in Golestan province, where this species exhibits a moderate level of habitat suitability. Therefore, the habitat suitability map reveals a clear geographic distribution pattern for *T*. *undulata*, indicating a strong presence in the south of the country and a lack of suitable habitats in other regions of the country.

Based on Table 2, we analyzed changes in the distribution of *T. undulata* under current and future climate conditions.

**Table 2:** Changes of suitable habitat categories based on climate scenarios in the future.

Climate scenario		Suitable habitat category							
		Unsuitable (Pixel)	Ratio (%)	Low (Pixel)	Ratio (%)	medium (Pixel)	Ratio (%)	High (Pixel)	Ratio (%)
Current	1970-2000	1712942	–	308755	–	123480	–	101578	–
SSP245	2030	1824910	6.5	251200	−18.6	127686	3.4	42959	−57.7
	2050	1843491	7.6	244523	−20.8	122654	−0.7	36087	−64.5
	2070	1862070	8.7	257968	−16.4	101981	−17.4	24736	−75.6
	2090	1874129	9.4	255035	−17.4	96769	−21.6	20822	−79.5
SSP585	2030	1809165	5.6	255546	−17.2	133971	8.5	48073	−52.7
	2050	1864371	8.8	248388	−19.6	107901	−12.6	26095	−74.3
	2070	1901410	11	258108	−16.4	77770	−37	9467	−90.7
	2090	1935347	13	262830	−14.9	46862	−62	1716	−98.3

#### Unsuitable (< 0.1) areas.

These areas are predicted to increase under both scenarios (SSP245 and SSP585) and across all future timeframes (2030, 2050, 2070, 2090). This means that in both scenarios and all future years, regions deemed unsuitable for *T. undulata* are predicted to expand. Under scenario SSP245, the unsuitable areas would increase by 6.5% by 2030 and up to 9.4% by 2090. Under scenario SSP585, the increase is slightly smaller initially (5.6% by 2030) but still reaches a 13% increase by 2090.

#### Low (0.1–0.4) suitable areas.

Under both scenarios, these areas are generally predicted to decrease across all future timeframes. Under scenario SSP245, the low suitable areas would decrease by 18.6% by 2030, and the decrease would continue at a varying rate, with a 17.4% reduction by 2090. Under scenario SSP585, a similar trend is observed, with a 17.2% decrease by 2030 and a 14.9% decrease by 2090.

#### Medium (0.4–0.7) suitable areas.

The trends for medium suitable areas are more variable. Under scenario SSP245, a slight increase of 3.4% is initially observed by 2030, but this is followed by a decrease in subsequent years, culminating in a 21.6% reduction by 2090. Under scenario SSP585, a larger initial increase of 8.5% is seen by 2030, but this trend reverses sharply, resulting in a significant 62% decrease by 2090.

#### High (> 0.7) suitable areas.

This category shows the most dramatic decreases. It’s projected that these high suitability areas will reduce substantially under both scenarios across all future timeframes. The most significant loss is seen in the year 2090 under both scenarios, where a decrease of 98.3% is predicted.

**Fig 5:**
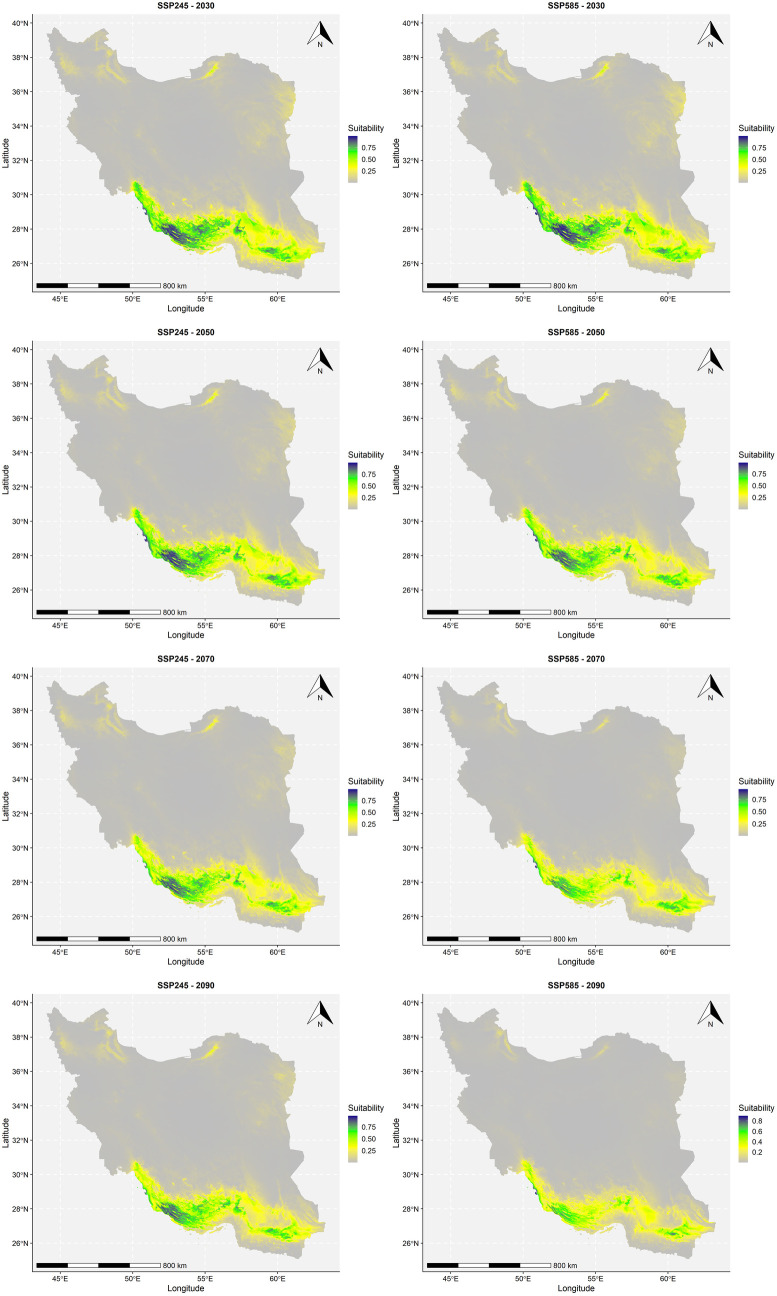
Habitat suitability of T. undulata. Map was generated using R 4.4.2 (https://www.r-project.org/).

In conclusion, the modeling predicts that suitable habitats for *T. undulata* (low, medium, and high suitability areas) will reduce in the future under both climate scenarios. At the same time, unsuitable habitats will expand (Fig 5). This suggests that future climatic conditions could negatively impact on the distribution of *T. undulata*, emphasizing the need for proactive measures to conserve this species.

### Response curve*s* of environmental variables

**Fig 6:**
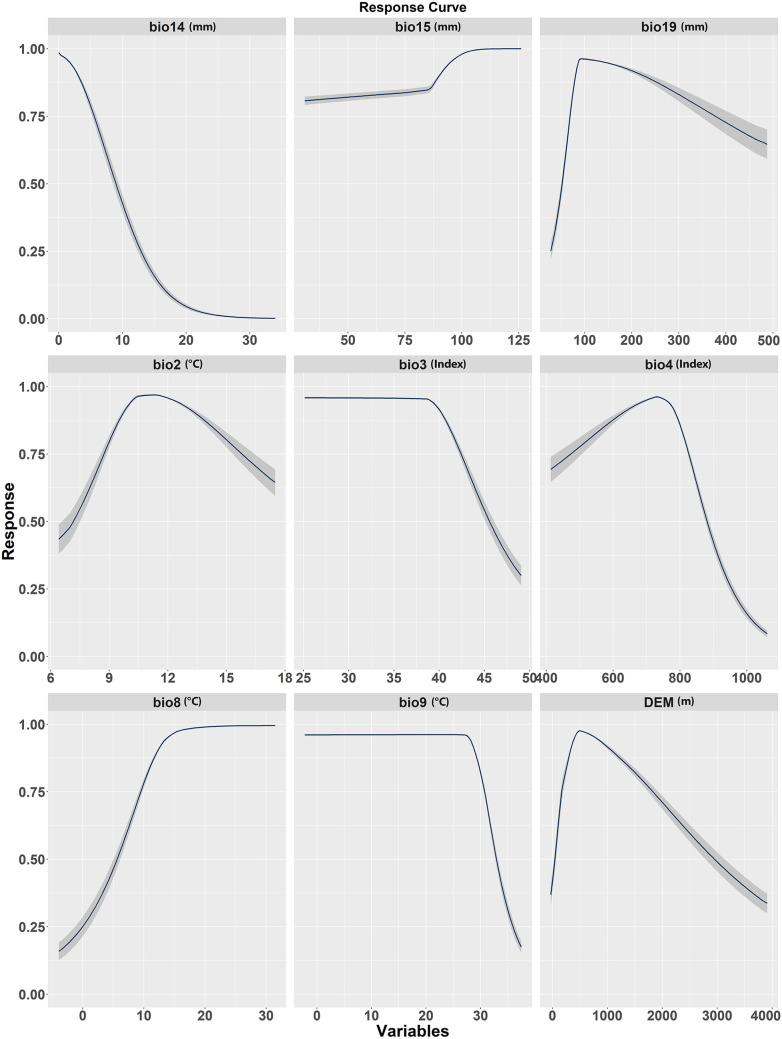
Response Curves of environmental variables.

The habitat suitability modeling of *T. undulata*, provided valuable insights through the analysis of response curves for several variables (Fig 6). The species showed preferences for certain environmental conditions, and these findings can inform conservation strategies and efforts. The variables analyzed included Bio4, Bio8, Bio15, Bio9, Bio19, Bio14, Bio2, Bio3, and DEM.

The species shows a preference for temperature ranges between −2–27°C in the driest quarter (Bio9) with declining suitability beyond 27°C. A moderate range of seasonal temperature variation (Bio4) is optimal, with a peak preference at a value of 720, beyond which suitability declines, underscoring its sensitivity to wide temperature fluctuations. *T. undulata* also favors warmer conditions during the wettest quarter (Bio8), indicative of its tropical adaptation, and exhibits a strong preference for environments with high precipitation seasonality (Bio15). The species shows optimum desirability for habitats with minimal diurnal temperature variation (Bio2), peaking at a value of 10.5. Regarding precipitation during the driest (Bio14) and coldest quarters (Bio19), habitat suitability decreases with increased wetness beyond certain thresholds, demonstrating a preference for drier conditions and a particular precipitation range during colder times. Elevational preferences (DEM) peak at intermediate levels, suggesting adaptability within a specific altitude range. Lastly, there is a declining habitat suitability with increased isothermality (Bio3), indicating a preference for low uniform temperature distribution.

## Discussion

### Considerations and limitations

Species distribution models (SDMs) are not without their limitations and underlying assumptions. For instance, they can be susceptible to biases arising from sampling methods, sample size, multicollinearity, and spatial autocorrelation. Additionally, while SDMs can be employed to project species distributions under future conditions, the uncertainty associated with these projections can be substantial. Furthermore, it is important to acknowledge that SDMs, particularly those focused primarily on climate variables, may not fully capture the influence of rapidly changing factors such as land use and human interventions. While our study utilizes SSPs scenarios to project future climate conditions, which partially cover these aspects by incorporating socioeconomic factors that drive land use patterns, they still represent a simplification of complex real-world dynamics. Therefore, future research could benefit from integrating land use change models and human impact assessments alongside climate projections to provide a more comprehensive understanding of potential species distributions. Despite these limitations, MaxEnt has become a widely used tool for habitat evaluation studies. However, it is important to note that MaxEnt’s presence-only models generally perform less effectively in species distribution modeling compared to presence-absence models, as absence data can provide valuable insights into species’ absence points. In contrast, MaxEnt has emerged as a superior tool compared to other species distribution models, especially in the context of evaluating the extensive uncertainties associated with modeling [28]. Therefore, the application of such modeling approaches is appropriate for Iran, considering the limited availability of data on both species distribution and climate [50]. The MaxEnt model's high AUC and TSS values affirm its robustness in delineating the potential habitat for *T. undulata*, providing us with a credible foundation to discuss the implications of our findings.

A common challenge in ecological studies, including species distribution modeling, is obtaining high-resolution climate data that accurately reflect the conditions experienced by organisms in their specific habitats. This is particularly true in regions with limited monitoring infrastructure, like Iran, where data on species occurrences and local climate are often scarce. Gridded climate datasets, such as WorldClim used in this study, help overcome this limitation by providing spatially continuous estimates of climate variables [33,51]. However, these datasets have inherent uncertainties due to factors such as input data quality and interpolation methods. Despite these uncertainties, gridded data are increasingly used in ecological [52,53] and agricultural research, as demonstrated by studies evaluating their utility in fields like crop modeling [54,55].

### Threats facing *T. undulata*

*T. undulata*, a versatile plant species significant for arid-rural communities, is currently under the threat of extinction due to rampant over-exploitation and inadequate conservation initiatives. Traditional propagation techniques relying on seeds are rendered ineffective as several obstacles such as human activities, environmental shifts, short seed viability, and improper harvest practices hinder the plant's natural regeneration process [20]. Compounding these issues is a lack of sustained conservation efforts from local populations. Concurrently, *T. undulata*’s population has been seeing a continual decrease within its natural habitats, primarily driven by numerous anthropogenic factors. Activities such as illegal deforestation, overharvesting, and habitat destruction significantly contribute to this alarming decline, emphasizing its fragile nature. Essentially, urgent and effective conservation measures are necessary to prevent further degradation of T*. undulata*’s population, highlighting the requirement for an immediate strategic approach in its preservation [15,18].

### Impact of climate change on *T. undulata* distribution

With temperature variables being the most influential predictors, any shifts in these conditions due to climate change could have pronounced effects on the species’ distribution. Temperature seasonality (bio4) emerged as the most significant variable, suggesting that *T. undulata* has a distinct phenological niche, tied closely to seasonal temperature variation. This specificity could imply a vulnerability to climate change, especially if temperature fluctuations become more erratic or deviate from the current seasonal patterns. The model's emphasis on temperature-related variables, including the mean temperature of the wettest (bio8) and the driest quarters (bio9), coupled with precipitation variables such as precipitation seasonality (bio15), underscores a potentially intricate environmental interplay that *T. undulata* depends on for its survival and reproduction. The species’ apparent preference for warmer temperatures during the wet quarters may hint at thermophilic reproductive or germination strategies, which are at odds with the projected increase in unsuitable areas under all climate scenarios (SSP245 and SSP585).

The findings indicate a pronounced decrease in areas of high suitability over the coming decades, with a 98% reduction by the year 2090 under the SSP585 scenario. Such drastic contractions could render *T. undulata* vulnerable to extinction, particularly because these regions represent the core of its climatic niche. On a regional level, the current distribution centered in the warmer southern provinces of Iran like Fars and Bushehr will face significant challenges as the unsuitable areas are predicted to increase. This geographic concentration suggests a limited adaptive capacity unless the species can migrate to or establish in new areas, which in itself is uncertain given the overall reduction in suitable habitats. The consistent increase in unsuitable habitats and the noteworthy reduction in optimal ones suggest that by 2090, *T. undulata* may be confined to extremely small pockets of suitable environments, if at all.

### Phenology and environmental variables

The flowers of this plant start to bloom from the beginning of April and this process continues until the end of May. The fruits of this plant were formed only in one region of the country and they existed until the end of May when the fruits started to fall as the weather got warmer. In some habitats, this species produces flowers twice a year. Once in early April and the second time in early November. The fall of leaves occurred in January and February (in Delfard of Jiroft habitat) and of Bushehr in July and August (in Shahnia and Moghdan habitat) [56]. Flowering time (April-May) coincides with the warmest and wettest period as preferred by the species (high Bio8). Moderate temperatures and precipitation during this time allow successful flowering. Fruiting in May requires maintaining temperature and humidity conditions. High temperatures by late May (Bio9 > 27) also lower habitat quality, causing fruit drop. Leaf drop in winter (Delfard) relates to low temperatures (Bio8). In contrast, summer leaf drop (Shahnia, Moghdan) is likely due to high temperatures (Bio9) and aridity causing moisture stress. Preference for moderate seasonal temperature variation (Bio4) is consistent with leaf retention in winter and summer, avoiding extreme cold or heat.

### Comparison with other studies

The present study, focusing on the impacts of climate change on *T. undulata* distribution in Iran, identified temperature seasonality (Bio4), mean temperature of the wettest quarter (Bio8), and precipitation seasonality (Bio15) as the primary drivers. This emphasis on temperature aligns with the findings of Arshad et al [18], who also highlighted the importance of Bio8 and Bio9 (mean temperature of the driest quarter) for the species in Pakistan. However, our study diverges by emphasizing precipitation seasonality, suggesting a greater sensitivity of *T. undulata* in Iran to fluctuations in rainfall patterns.

Mathur and Mathur [15], conducting a global assessment, identified November wind speed and isothermality (Bio3) as the principal factors influencing the species’ distribution, alongside precipitation of the wettest quarter (Bio16) and mean temperature of the warmest quarter (Bio10). However, the inclusion of wind speed in their model underscores the potential impact of additional climatic factors at a global scale, which was not used in our regional study.

Mathur and Mathur [14], focusing on India, also emphasized the role of temperature and precipitation, particularly annual precipitation and maximum temperature of warmest month. Precipitation seasonality was identified as the third most important variable for both current conditions and the 2070 projections, which aligns with the findings of our study. This further supports the critical role of these factors in shaping *T. undulata*’s distribution. However, their study also considered soil properties, habitat heterogeneity indices, and land-use land cover, revealing a facilitative rather than limiting effect of these non-climatic variables on the species’ distribution in India.

These discrepancies in variable importance and the inclusion of unique factors in each study highlight the influence of specific environmental contexts and habitat characteristics on *T. undulata*’s distribution across different regions and scales. The variation in methodological approaches, such as the use of Shared Socioeconomic Pathways (SSPs) in our study versus Representative Concentration Pathways (RCPs) in Arshad et al [18], and the incorporation of ensemble modeling in Mathur and Mathur [15], further contributes to the observed differences.

Our national-scale analysis provides a broad overview of *T. undulata*’s climate-driven suitability, but finer-scale regional variations and microclimates, not fully captured at our 1 km² resolution, influence local presence. Factors like slope, aspect, soil, and water availability can create more or less favorable microclimates within generally suitable regions, potentially forming refugia. Future research with finer-resolution data is needed to understand these microclimatic influences.

While our study provides a robust assessment of *T. undulata*’s vulnerability to climate change in Iran, it is crucial to acknowledge that the species’ response might vary across its range due to local adaptations and environmental heterogeneity. Future studies incorporating a wider range of predictors, including biotic interactions and disturbance regimes, could enhance our understanding of the species’ ecological niche and inform more targeted conservation strategies. Additionally, long-term monitoring and experimental studies are needed to validate the model predictions and assess the species adaptive capacity in the face of ongoing climate change.

### Conservation strategies for *T. undulata* in a changing environment

Evidence strongly suggests that *T. undulata* is projected to face a constricting range in Iran due to climate change, with significant implications for its conservation status. The spatiotemporal dynamics observed in *T. undulata* highlight the broader need for robust predictive models to inform adaptive management strategies under climate change. Considering these factors, targeted conservation strategies can be developed. Areas featuring moderate seasonal temperature fluctuations, warmer temperatures during the wettest quarter, high precipitation seasonality, relatively small daily temperature fluctuations, moderate temperatures during the driest quarter, and intermediate elevations should be prioritized for conservation efforts. Integrating these findings of habitat preferences into conservation plans is crucial to enhance the long-term survival of *T. undulata* under climate change and other threats.

In light of the predicted decline in habitat suitability, specific and actionable conservation measures are needed. The establishment of protected areas should be prioritized, focusing on regions projected to remain suitable under future climate scenarios, especially those currently harboring significant populations of *T. undulata*. Given the potential for range shifts, carefully managed assisted migration or translocation programs could be considered, although these would require thorough ecological risk assessments. Habitat restoration efforts should be implemented in areas where habitat has been degraded but that are predicted to be climatically suitable in the future, potentially involving active planting of *T. undulata* and the removal of invasive species. Additionally, the creation of habitat corridors can help connect fragmented *T. undulata* populations, facilitating genetic exchange and promoting overall species resilience. A multi-faceted approach involving both in-situ and ex-situ conservation is vital. Ex-situ conservation programs, such as seed banks and botanical gardens, can play a crucial role in safeguarding the genetic diversity of *T. undulata* and provide material for future restoration efforts. Concurrently, engaging local communities through the promotion of sustainable land management practices and raising awareness about the ecological and economic importance of *T. undulata* can foster stewardship and encourage active participation in conservation.

Future research should focus on long-term ecological monitoring to track changes in the species’ distribution and correlate these shifts with climatic variables, and delving into the genetic diversity will inform on the species’ adaptive potential. Additionally, testing the physiological resilience of *T. undulata* to anticipated extreme climatic events is crucial. Predictive models must be continually updated, factoring in land-use alterations to refine projections.

Finally, legal measures should enshrine these efforts in policy, aligning land-use practices with preservation goals. Collaboration between research and protection measures is the cornerstone of effective conservation, offering a significantly better chance of mitigating the adverse effects of climate change on *T. undulata* and ensuring its persistence into the future.

## Conclusion

This study highlights the precarious status of the rare arid ecosystem species, *T. undulata*, in the face of climate change. Our predictive models show its suitable habitat will shrink due to shifts in temperature and precipitation patterns. This is especially concerning in regions of current high suitability, which face significant reductions under both SSP245 and SSP585 climate scenarios. Temperature seasonality, the mean temperature of the wettest quarter, and precipitation seasonality are the key climate factors influencing *T. undulata’s* distribution. Elevation also plays a notable role, further demonstrating the complexity of its habitat needs.

We advocate for integrating predictive habitat modeling into conservation efforts. This provides crucial insights for proactive measures such as preserving key habitats, restoring degraded areas, and establishing protected zones. To protect biodiversity hotspots like Iran, adaptive management is vital, using policies that promote both sustainable land use and climate change mitigation.

Future research should focus on long-term ecological monitoring, exploring *T. undulata’s* genetic diversity, understanding its interactions with other species, and assessing its resilience to extreme weather events. This information will further strengthen and refine our predictive models.

Conservation must be multifaceted, including habitat restoration, ecological corridors, ex-situ collections (e.g., seed banks), and community involvement. Legal frameworks are critical to align land use with preservation goals. By uniting research and conservation action, we can significantly improve our chances of safeguarding *T. undulata* from the adverse effects of climate change.

## Supporting information

S1 File R scripts. R scripts used MaxEnt modeling.
(RAR)

S2 File Occurrence points. occurrence points used in modeling. (CSV)
